# Hyperspectral Prediction Models of Chlorophyll Content in *Paulownia* Leaves under Drought Stress

**DOI:** 10.3390/s24196309

**Published:** 2024-09-29

**Authors:** Yamei Zhang, Guangxin Ru, Zhenli Zhao, Decai Wang

**Affiliations:** College of Forestry, Henan Agricultural University, Zhengzhou 450046, China; zym@henau.edu.cn (Y.Z.);

**Keywords:** *Paulownia* seedlings, drought stress, preprocessing, leaf position, chlorophyll content, partial least squares

## Abstract

This study explored the quantitative inversion of the chlorophyll content in *Paulownia* seedling leaves under drought stress and analyzed the factors influencing the chlorophyll content from multiple perspectives to obtain the optimal model. *Paulownia* seedlings were selected as the experimental materials for the potted water control experiments. Four drought stress treatments were set up to obtain four types of *Paulownia* seedlings: one pair of top leaves (T1), two pairs of leaves (T2), three pairs of leaves (T3), and four pairs of leaves (T4). In total, 23 spectral transformations were selected, and the following four methods were adopted to construct the prediction model, select the best spectral preprocessing method, and explore the influence of water bands: partial least squares modeling with all spectral bands (all-band partial least squares, AB-PLS), principal component analysis partial least squares (PCA-PLS), correlation analysis partial least squares (CA-PLS), correlation analysis (water band) partial least squares, ([CA(W)-PLS]), and vegetation index modeling. Based on the prediction accuracy and the uniformity of different leaf positions, the optimal model was systematically explored. The results of the analysis of spectral reflectance showed significant differences at different leaf positions. The sensitive bands of chlorophyll were located near 550 nm, whereas the sensitive bands of water were located near 1440 and 1920 nm. The results of the vegetation index models indicate that the multiple-index models outperformed the single-index models. Accuracy decreased as the number of indicators decreased. We found that different model construction methods matched different optimal spectral preprocessing methods. First derivative spectra ($R^{\prime }$) was the best preprocessing method for the AB-PLS, PCA-PLS, and CA-PLS models, whereas the inverse log spectra (log(1/R)) was the best preprocessing method for the CA(W)-PLS model. Among the 14 indices, the green normalized difference vegetation index (GNDVI) was most correlated with the chlorophyll content sensitivity indices, and the water index (WI) was most correlated with the water sensitive indices. At the same time, the water band affected the cross validation accuracy. When characteristic bands were used for modeling, the cross validation accuracy was significantly increased. In contrast, when vegetation indices were used for modeling, the accuracy of the cross validation increased slightly but its predictive ability was reduced; thus, these changes could be ignored. We found that leaf position also affected the prediction accuracy, with the first pair of top leaves exhibiting the worst predictive ability. This was a bottleneck that limited predictive capability. Finally, we found that the CA(W)-PLS model was optimal. The model was based on 23 spectral transformations, four PLS construction methods, water bands, and different leaf positions to ensure systematicity, stability, and applicability.

## 1. Introduction

Drought is the most common abiotic stressor in plants, and the occurrence of drought stress triggers active responses that affect various physiological and biochemical characteristics of plants. Chlorophyll is an important biochemical indicator of plant growth, and its content is critical for identifying plant drought stress, leaf photosynthesis capacity, and growth status at various developmental stages [1]. Therefore, chlorophyll content is often used as an effective indicator of plant growth health and stress resistance when plants are under drought stress [2].

In recent years, hyperspectral data have been widely used for the quantitative inversion of plant physiological parameters. The inverted plant physiological parameters have mainly focused on nitrogen, chlorophyll, and water content [3,4,5], and the research scales were mainly divided into leaf and canopy scales. At the leaf scale, model research on chlorophyll content has mainly focused on two aspects: (1) obtaining position, amplitude, area, and other information based on the spectral curve [6], analyzing the spectral characteristic responses, and making predictions; and (2) inverting chlorophyll content based on the spectral reflectance, including direct modeling using all-band data [7], dimensionality reduction modeling using all-band data [8], modeling by selecting feature bands [1], and vegetation index modeling [9]. Direct modeling using all-band data is the most direct and simple method; however, due to the large amount of data in all bands, a certain degree of information overlaps between bands. Therefore, dimensionality reduction or the selection of characteristic bands is usually performed before model construction. The methods of direct modeling using all-band data, dimensionality reduction modeling using all-band data, and modeling by selecting characteristic bands are generally consistent. Common methods include partial least squares (PLS), neural networks, support vector machines, and random forest algorithms. Spectral preprocessing is often required before modeling to accurately extract the characteristic information and improve the prediction model. There are many spectral preprocessing methods, such as first-order differential and multiplicative scatter correction (MSC) [4,10,11]. However, not all methods can improve the modeling effect; thus, it is necessary to select the best spectral preprocessing method. Vegetation index modeling includes both multi-vegetation index modeling and single-vegetation index modeling. Multi-vegetation index modeling is consistent with the other three modeling methods, whereas single-vegetation index modeling commonly uses regression methods, such as linear, exponential, and power functions.

The prediction between hyperspectral reflectance and chlorophyll content at the leaf scale is mainly conducted on crops such as wheat [10], corn [12], rice [13], and cotton [14]. It is widely applied in forestry trees, including *Poplar* [1], *Eucalyptus* [15], *Quercus aliena* var. *acuteserrata* Maxim. [16], P*terocaryastenoptera* [8], *Apple* [17], and *Citrus* [18]. However, research on *Paulownia* has not yet been reported. The hyperspectral prediction of chlorophyll content involves spectral preprocessing, spectral data dimensionality reduction, and modeling. There is no unified consensus on how to achieve a high-precision prediction for different plant types, so this remains a research question that requires further exploration. Conducting research on *Paulownia* will further enrich the supporting data.

Research on *Paulownia* under drought stress has focused mainly on growth [19], the physiological and biochemical aspects [20], chlorophyll fluorescence [21], and genes [22]. Under drought stress, the relative water and chlorophyll contents of diploid *Paulownia* seedlings and their tetraploids decreased [20]. The relative water content and chlorophyll unit area mass of 3-year-old *Paulownia fortunei* and *Paulownia elongata* leaves also gradually decreased [23]. The relative water content and photosynthetic rate of *Paulownia catalpifolia* seedlings leaves gradually decreased [21]. A decrease in leaf water content can affect normal photosynthesis in plants, destroy the photosynthetic apparatus and photosynthetic reaction system, and lead to a decrease in the chlorophyll content. Therefore, the water content can also affect the chlorophyll content [13], and the leaf spectral reflectance is mainly affected by the leaf chlorophyll content, cell structure, and water content. Based on these results, this study attempts to predict the chlorophyll content by taking water bands into consideration, which has not been previously reported in *Paulownia* studies.

The study of hyperspectral prediction models for chlorophyll content in plant leaves has included model research without considering the leaf position and vertical distribution layers [8,14], and model research targeting different leaf positions and vertical distribution layers. The research on chlorophyll content in wheat, based on different leaf positions and vertical distribution layers, also showed that the prediction accuracy of the same prediction model (PLS) was different [7], and that the optimal estimation models screened by different prediction models (PLS, support vector machine, random forests, and back propagation neural network) were also different [24], as was the cross validation accuracy of vegetation index modeling [25]. Different leaf positions and vertical distribution layers affect the selection of the best model. Therefore, this study considered the predictive ability of different leaf positions.

**Figure 1:**
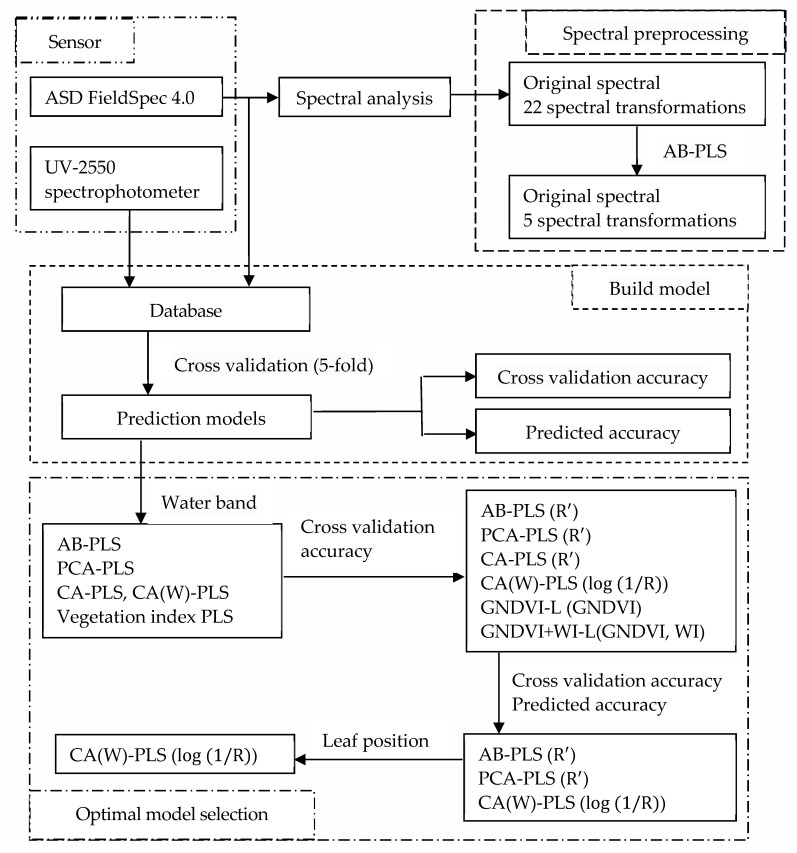
The research ideas and technical procedures.

In this study, *Paulownia* seedlings were used as experimental subjects to investigate the hyperspectral prediction of chlorophyll content under drought conditions (Figure 1). Firstly, the characteristics of the spectral curves of *Paulownia* leaves were analyzed to clarify their performance at different leaf positions and water-sensitive bands. Secondly, 23 spectral transformations were selected from which six spectral preprocessing methods were selected for modeling. Four modeling methods were adopted: direct modeling using all-band data, all-band dimensionality reduction modeling based on principal component analysis, feature band selection modeling based on correlation analysis, and vegetation index modeling. Four PLS models were constructed, and optimal models of the different modeling methods and their corresponding spectral preprocessing methods or vegetation indices were screened according to accuracy evaluation indicators, with a preliminary exploration of the impact of water bands. Finally, based on the prediction accuracy and uniformity of the different leaf positions, the optimal prediction model was systematically explored. Using ground-measured data, the chlorophyll content was predicted to provide technical support in research using aerial and satellite data and to provide a reference for the cultivation management and drought assessment of *Paulownia* seedlings.

## 2. Materials and Methods

### 2.1. Experimental Design

Seeds from *P. tomentosa* (Thunb.) Steud × *P. fortunei* (Seem.) Hemsl 33 were collected and planted at the Science and Education Park of Henan Agricultural University in Zhengzhou, Henan Province (113°35′ E, 34°51′ N) in the following year. Seeds were sown in March, and healthy seedlings with similar growing consistency, on which the fourth pair of top leaves had just fully unfolded, were selected and transplanted into plastic pots (24 cm × 20 cm × 24 cm) in May. Each pot contained one seedling and 5 kg of air-dried soil (dried under natural conditions and sifted through a 10 mm sieve). In July, *Paulownia* seedlings with consistent growth and five pairs of leaves and three pairs of fallen leaves with fully unfolded top leaves were selected for the drought stress experiments. The maximum water-holding capacity of the soil was calculated as the ratio between the weight of the water in the pot and the weight of the dry soil in the pot. The entire pot, including the plant, pot, and soil, was submerged in water for 12 h, after which the excess water was allowed to drain. We obtained the soil with the maximum water-holding capacity and then weighed it to determine its weight for all soil (wet soil weight). Wet soil was oven-dried at 105 °C for 48 h to determine its dry mass (dry soil weight). The water weight in the pot was the wet soil weight minus the dry soil weight [26]. Four treatments were set up with soil water contents of 25–30%, 40–45%, 55–60%, and 70–75% of the maximum water-holding capacity. The experiment was terminated when only one pair of top leaves remained on the seedlings under the most severe drought stress conditions. At this point, four types of *Paulownia* seedlings were collected, corresponding to the four treatments: one pair of top leaves (T1, 25 seedlings), two pairs of leaves (T2, 25 seedlings, with some having only one leaf in the second pair), three pairs of leaves (T3, 25 seedlings, with some having only one leaf in the third pair), and four pairs of leaves (T4, 25 seedlings, with some having only one leaf in the fourth pair). Starting from the first pair of fully unfolded leaves at the top of the *Paulownia* seedlings, they were sequentially referred to as the first pair of top leaves, second pair of leaves, third pair of leaves, and fourth pair of leaves from top to bottom.

### 2.2. Indicator Measurements and Methods

#### 2.2.1. Leaf Reflectance

Since the chlorophyll value at the leaf base of the *Paulownia* seedlings was closest to the average chlorophyll value of the leaf [27], points of measurement were selected in the leaf base (avoiding the leaf veins), which were symmetrically located on both sides of the main leaf veins, to measure all unfolded leaves of the *Paulownia* seedlings. Reflectance measurements were conducted using the ASD FieldSpec 4.0 (Analytical Spectral Devices, Boulder, CO, USA). The instrument’s built-in light source and leaf clip were used to obtain 10 sets of spectral data at each measurement point, and 20 sets of spectral data were obtained from each leaf. The average value was taken as the spectral value of the leaf. The resampling interval was 1 nm, and the waveband range was 350–2500 nm.

#### 2.2.2. Chlorophyll Content

Centered on the position determined by spectrometry, 0.3 g of leaves were cut from both sides of the main leaf veins, resulting in a total of 0.6 g. After being cut into pieces and ground, the pieces were divided into three portions. One portion (0.2 g) was mixed with an 80% acetone extraction solution to a fixed volume of 25 mL. The leaves were then placed in a dark place for extraction until they turned completely white. We used the Shimadzu UV-2550 spectrophotometer (Shimadzu, Kyoto, Japan). The A_663_ and A_646_ values were measured at 663 and 646 nm, respectively. Chlorophyll content was calculated using the following formula [28,29]:

(1) $$C=\frac{V\times (C_{a}+C_{b})}{1000M}=\frac{V\times (17.32A_{646}+7.18A_{663})}{1000M}$$

(2) $$C_{a}=12.21A_{663}-2.81A_{646}$$

(3) $$C_{b}=20.13A_{646}-5.03A_{663}$$

where *C* is the chlorophyll content (mg/g), *V* is the volume of the extraction solution (mL), $C_{a}$ is chlorophyll *a* (mg/L), $C_{b}$ is chlorophyll *b* (mg/L), $A_{646}$ is the absorbance value at 646 nm, $A_{663}$ is the absorbance value at 663 nm, and *M* is the fresh leaf mass (mg).

### 2.3. Preprocessing the Original Reflectance Data

Spectral preprocessing can alter the shape of the spectrum and the trend of the spectral curve, thereby affecting the accuracy of the prediction model. Spectral preprocessing was performed using 22 commonly used methods to transform the original spectrum, including the following: first derivation ($R^{\prime }$), first derivation standard normal variate (FD-SNV), first derivation multiplicative scatter correction (FD-MSC), second derivation (R″), square root ($\sqrt{R}$), logarithm (log⁡R), reciprocal (1/R), first derivation of the square root (($\sqrt{R}$)′), first derivation of the logarithm ((log⁡R)′), first derivation of the reciprocal ((1/R)′), logarithm of the reciprocal (log(1/R)), first derivation of the logarithm of reciprocal ((log(1/R))′), logarithm of the square root ($log\sqrt{R}$), first derivation of the logarithm of the square root (($log\sqrt{R}$)′), reciprocal of the logarithm (1/log R), first derivation of the reciprocal of logarithm (${\left(1/logR\right)}^{\prime }$), square root of the reciprocal ($\sqrt{1/R}$), first derivation of the square root of the reciprocal ($\sqrt{1/R}$)’), standard normal variate (SNV), and MSC [4,10,11,29,30].

### 2.4. Regression Model

In total, 23 spectral transformations (the original spectrum and 22 preprocessing types of the original spectrum) were adopted. First, all bands were selected, all bands had a dimension reduction by principal component analysis, feature bands were selected by Pearson correlation analysis, and commonly used vegetation indices were obtained. Then, PLS modeling was performed. The PLS model adopted the regression modeling of a single dependent variable to multiple independent variables, which was suitable for the all-band spectrum with a particularly large number of variables and a few spectral indices and spectral principal components. The simplest form is a linear regression [31], which is expressed as follows:

(4) $$y=a_{0}+a_{1}x_{1}+a_{2}x_{2}+\cdots +a_{n}x_{n}$$

where *y* is the chlorophyll content, $a_{0}$ is the intercepts of the regression coefficient, $a_{i}$ is the regression coefficient, and $x_{i}$ is the independent variable from 1 to *n*.

Of all the *Paulownia* seedlings, 478 leaves (100 *Paulownia* seedlings) were used for the PLS model. The 5-fold cross validation was adopted to evaluate the accuracy of the model. The evaluation indicators of the cross validation accuracy and the predicted accuracy included the determination coefficient (R^2^) and the root mean square error (RMSE).

In this study, ViewSpec Pro 6.0 was used for the spectral data processing; R 3.8 was used for the principal component analysis, correlation analysis, partial spectral data preprocessing, and plotting; and Unscrambler 9.7 was used for the modeling.

## 3. Results

### 3.1. Analysis of the Spectral Curve Characteristics

**Figure 2:**
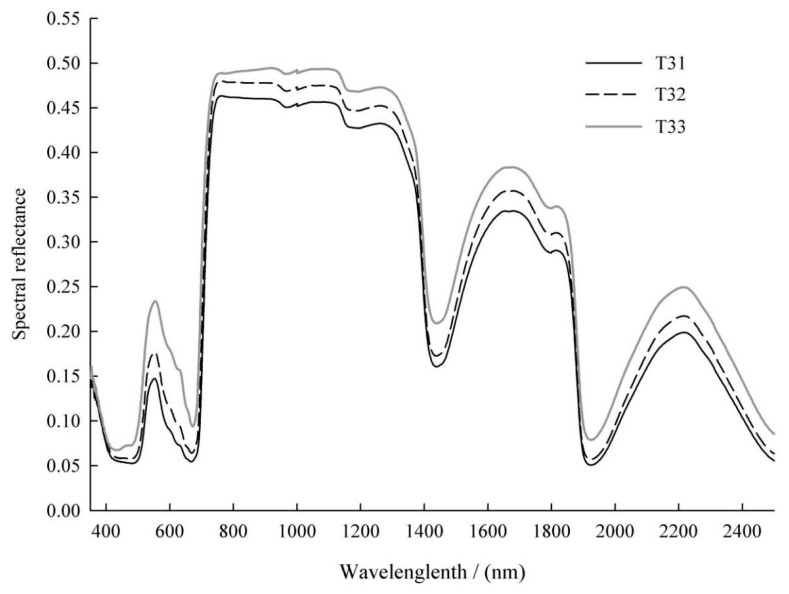
Mean spectral reflectance curves of T3 at different leaf positions.

**Figure 3:**
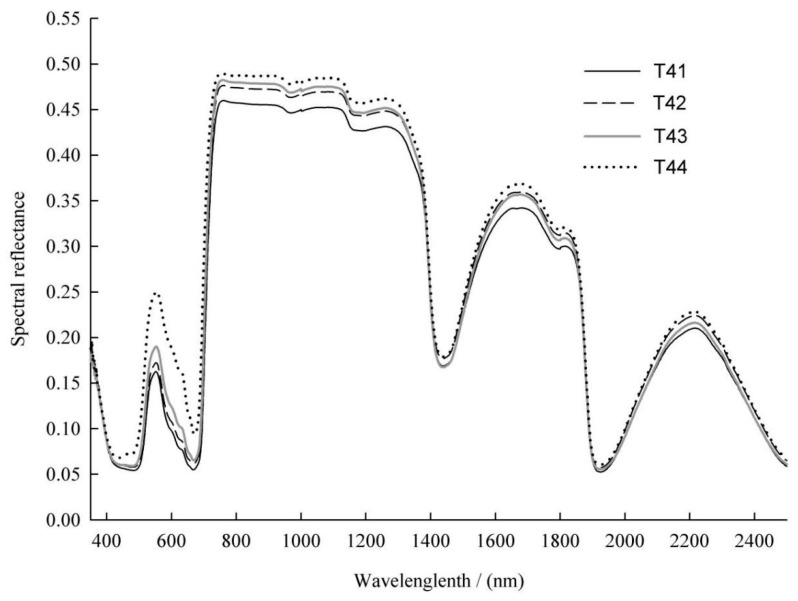
Mean spectral reflectance curves of T4 at different leaf positions.

Under drought stress, obvious differences were observed in the average spectral reflectance of the leaves at different leaf positions on T3, and the pattern was consistent (Figure 2). In the vicinity of the chlorophyll-sensitive green light reflection peak at 550 nm and the water-sensitive bands at 1440 and 1920 nm, the reflectance was in the following order: T33 (the third pair of leaves on T3) > T32 (the second pair of leaves on T3) > T31 (the first pair of top leaves on T3). Some differences were observed in the average spectral reflectance of the leaves at different leaf positions on T4, but the changes were more complex than those on T3 (Figure 3). The difference in the vicinity of the green light reflection peak at 550 nm was more significant, showing that T44 (the fourth pair of leaves on T4) > T43 (the third pair of leaves on T4) > T42 (the second pair of leaves on T4) > T41 (the first pair of top leaves on T4). In the vicinity of the water absorption peak at 1440 nm, the performance was T44 > T42 > T41 > T43; in the vicinity of the water absorption peak at 1920 nm, the performance was T44 > T42 > T43 > T41. Therefore, under drought stress, there are differences in the average spectral reflectance among the leaves at different leaf positions on *Paulownia* seedlings.

The average spectral reflectance of the first pair of apical leaves was relatively low, whereas that of the leaves at the lowermost leaf position was relatively high (Figure 2 and Figure 3). In the vicinity of the chlorophyll-sensitive green light reflection peak near 550 nm, the difference in spectral reflectance was most obvious, indicating a significant difference in chlorophyll content. In the vicinity of the water-sensitive bands near 1440 and 1920 nm, the spectral reflectance of the leaves at the lowermost leaf position was highest, indicating the lowest water content. These results indicate that the average spectral reflectance curve reflects the spectral response of chlorophyll and water content in leaves to drought stress.

### 3.2. PLS Modeling

#### 3.2.1. All-Band PLS (AB-PLS)

**Figure 4:**
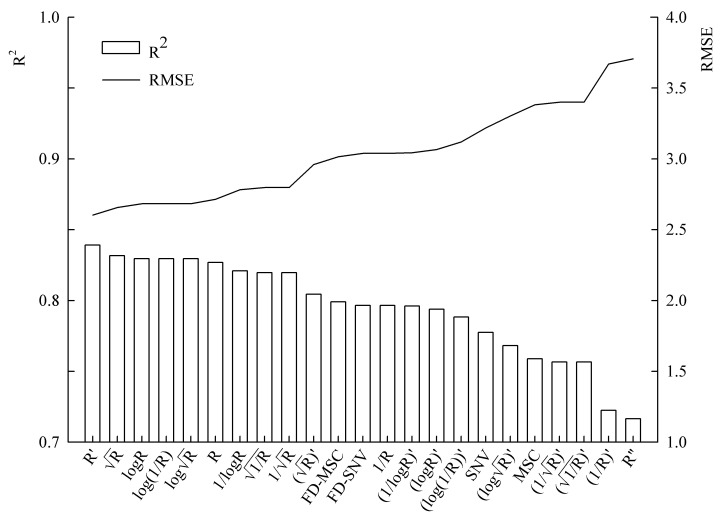
Cross validation accuracy for the 23 spectral transformations.

A total of 23 spectral transformations were used for AB-PLS modeling. According to the cross validation accuracy indicators R^2^*_CV_* and RMSE*_CV_*, six spectral preprocessing methods that achieved a cross validation accuracy greater than or equal to that of reflectivity (R) were selected: $R^{\prime }$, $\sqrt{R}$, log⁡R, $log(1/R),log\sqrt{R}$, and R. Among them, $R^{\prime }$ was the best spectral preprocessing method with the highest accuracy (R^2^*_CV_* = 0.8329, RMSE*_CV_* = 2.6422) (Table 1). The model established with R had the lowest accuracy (R^2^*_CV_* = 0.8268, RMSE*_CV_* = 2.7154) (Table 1). The accuracy difference between the models established with $R^{\prime }$ and R was relatively small, and the accuracy of the models established with log⁡R, log(1/R), and $log\sqrt{R}$ was consistent. The cross validation accuracies of the six spectral preprocessing methods were all relatively high and similar (Figure 4).

#### 3.2.2. Principal Component Analysis PLS (PCA-PLS) 

Principal component analysis (PCA) was the most selected dimensionality reduction method. It can transform a large amount of spectral information into a few comprehensive indicators, not only retaining most of the information from the original spectrum but also greatly reducing the dimensionality of the data. The results of the PCA were primarily evaluated using two indicators: the cumulative variance contribution rate, and the number of principal components. The cumulative variance contribution rate represented the strength of the information about the original variables that was contained in the principal components. The higher the cumulative variance contribution rate, the stronger the information about the original variables that was contained. The number of principal components referred to the quantity of principal components that had been extracted. The six preprocessing methods were used to extract the principal components according to the principle that the eigenvalue should be greater than 1, and PLS was performed using the obtained principal components. According to the PCA results, the cumulative contribution rate of $R^{\prime }$ was the lowest (96.08%) (Table 2), with the largest number of principal components (170), indicating the worst dimensionality reduction ability. The cumulative contribution rates of log⁡R, log(1/R), and $log\sqrt{R}$ were all 99.82%, and the number of principal components was 9 for each, indicating that their dimensionality reduction abilities were consistent. From the perspective of modeling accuracy, the model established with $R^{\prime }$ had the highest accuracy, with R^2^*_CV_* having the maximum value (0.8769) and RMSE*_CV_* having the minimum value (1.9637). The cross validation accuracies of log⁡R, log(1/R), and $log\sqrt{R}$ were consistent. In contrast, the cross validation accuracy established with R was the lowest (R^2^*_CV_* = 0.8293, RMSE*_CV_* = 2.6857). The difference in cross validation accuracy between $R^{\prime }$ and R was not significant, and in both models, the R^2^*_CV_* values were above 0.82, indicating that the cross validation accuracies of the six spectral preprocessing methods were relatively high.

#### 3.2.3. Correlation Analysis PLS (CA-PLS)

Pearson correlation analysis was conducted to examine the correlation between the six spectral reprocessing methods and the chlorophyll content. Based on the principle that the larger the absolute correlation value the better, 10 feature bands (Table 3) were selected and used to establish the CA-PLS models. The correlation between $R^{\prime }$ and the chlorophyll content was strongest. Among the 10 selected bands, the minimum correlation value of $R^{\prime }$ was 0.7981, which was greater than the maximum values of the other five spectral preprocessing methods. The bands selected using $R^{\prime }$ were the continuous red-edge bands from 738 to 747 nm, with 744 nm having the strongest correlation. Bands selected using $\sqrt{R}$, log⁡R, log(1/R), and $log\sqrt{R}$ were all continuous green light bands from 544 to 553 nm, and the bands selected using R were the continuous green light bands from 545 to 554 nm, with only 554 nm being different.

After performing the pearson correlation analysis for the six spectral preprocessing methods, six feature bands were selected based on the principle that the larger the absolute correlation value the better. According to the water absorption peaks at 1440 nm and 1920 nm in the average spectral reflectance curve of the *Paulownia* seedlings’ leaves at different leaf positions, the water absorption ranges of 1390–1490 nm and 1870–1970 nm were selected, then the two bands with the strongest correlation were selected from each range, totaling four bands. Ten feature bands were selected in two separate rounds for the PLS modeling (correlation analysis (water band) PLS (CA(W)-PLS)). As shown in Table 3, the absolute values of the correlation coefficients between the water absorption bands and the chlorophyll content were relatively low, with a maximum of 0.1052 and a minimum of 0.0166. The water bands selected using log⁡R, log(1/R), and $log\sqrt{R}$ were all 1464, 1465, 1933, and 1944 nm, with the absolute correlation values being 0.0743, 0.0744, 0.0632, and 0.0669, respectively. This indicated that the positions of the bands and degrees affected by the three pretreatments near the water absorption peak were consistent. In the 1390–1490 nm band range, the maximum correlation between R and the chlorophyll content was 0.0819, which was higher than those of the other five spectral preprocessing methods. In the 1870–1970 nm band range, the maximum correlation between R and the chlorophyll content was 0.1052, which was also higher than those of the other five spectral preprocessing methods. This suggests that all the other five spectral preprocessing methods weakened the influence of the bands near the water absorption peaks to some extent.

According to Table 4, among the CA-PLS models established using the six spectral preprocessing methods, the model established using $R^{\prime }$ had the highest accuracy and the best spectral preprocessing method (R^2^*_CV_* = 0.7300, RMSE*_CV_* = 3.6133). In contrast, the model established using the R method had the lowest accuracy (R^2^*_CV_* = 0.6901, RMSE*_CV_* = 3.9042). However, the difference in accuracy between the best and worst models was not significant. In the CA(W)-PLS models, the accuracy of the models built using log⁡R and log(1/R) was almost the same (the maximum difference in R^2^*_CV_* and in RMSE*_CV_* were 0.0001). However, log(1/R) ranked as the best spectral preprocessing method, with a slightly higher modeling accuracy (R^2^*_CV_* = 0.8094, RMSE*_CV_* = 2.9085). The model with the lowest accuracy was built using $R^{\prime }$ (R^2^*_CV_* = 0.7937, RMSE*_CV_* = 3.0666). A relatively large difference in accuracy was observed between the best and worst models in CA(W)-PLS.

The cross validation accuracy of each of the CA(W)-PLS models was higher than that of the corresponding CA-PLS models, indicating that considering the feature bands of water could improve the cross validation accuracy. The improvement in accuracy was more significant for the models built using log⁡R and log(1/R) whereas the increase was minimal for the model built using $R^{\prime }$. It was worth noting that, although $R^{\prime }$ was the best spectral preprocessing method in the CA-PLS models, it became the worst when water was considered in the CA(W)-PLS models. This suggests that the best spectral preprocessing method changes depending on the feature bands used for modeling.

#### 3.2.4. Vegetation Index PLS

A total of 14 spectral indices were selected: 10 commonly used chlorophyll-sensitive vegetation indices and 4 commonly used water-sensitive indices. PLS modeling was performed using 10 vegetation indices (vegetation index partial least squares (10VI-PLS)). PLS modeling was also conducted using 10 vegetation indices and 4 water indices (vegetation index and water index partial least squares (10VI + 4WI-PLS)). Pearson correlation analysis was performed between the 14 indices and the chlorophyll content values (Table 5). Based on the principle of selecting the indices with the largest absolute correlation value, six vegetation indices (GNDVI, CIgreen, RVI, CI red edge, VOG3, and RNDVI) were selected for PLS modeling (vegetation index PLS (6VI-PLS)). Then, four water indices were added for PLS modeling (vegetation index and water index PLS (6VI- + 4WI-PLS)). Three vegetation indices with relatively large absolute correlation values were selected for PLS modeling (GNDVI linear (GNDVI-L), CIgreen linear (CIgreen-L), and RVI linear (RVI-L)), and then the water index (WI) with the largest absolute value of correlation was added for further modeling (GNDVI + WI-linear (GNDVI + WI-L), CIgreen + WI-linear (CIgreen + WI-L), and RVI + WI-linear (RVI + WI-L)) (Table 6).

Based on the models established using the vegetation indices alone compared with those using both the vegetation and water indices, the cross validation accuracy increased as the number of indicators increased. However, because different spectral indices had different effects on the model, identifying the best model and its corresponding modeling indices was challenging. Among the single-index models, GNDVI-L demonstrated the highest accuracy among the 10 vegetation indices, establishing it as the best single modeling indicator.

The models established using multiple indices outperformed the models established using a single index. Accuracy decreased as the number of indicators decreased. The accuracy of the 10VI-PLS model (R^2^*_CV_* = 0.8194, RMSE*_CV_* = 2.8000) was higher than that of the 6VI-PLS model (R^2^*_CV_* = 0.7984, RMSE*_CV_* = 3.1352), and both outperformed the single-index models GNDVI-L, GI_green_-L, and RVI-L. The accuracy of the 10VI + 4WI-PLS model (R^2^*_CV_* = 0.8246, RMSE*_CV_* = 2.7410) was higher than that of the 6VI + 4WI-PLS model (R^2^*_CV_* = 0.7989, RMSE*_CV_* = 3.0977), and both outperformed the single-index models GNDVI + WI-L, GI_green_ + WI-L, and RVI + WI-L.

After adding the WI, the accuracy of both the multiple-index and single-index models increased, although by a small margin. Compared with the 10VI-PLS model, the 10VI + 4WI-PLS model showed the largest variation, with R^2^*_CV_* increasing by 0.0052 and RMSE*_CV_* decreasing by 0.0590. Compared with the GI_green_-L and the RVI-L models, the R^2^*_CV_* values of the GI_green_ + WI-L and RVI + WI-L models did not change, and the RMSE*_CV_* only changed by 0.0001 and 0.0004. The change was so small that it indicated that the WI contributed very little to the cross validation accuracy. Therefore, when predicting the chlorophyll content in the leaves of *Paulownia* seedlings, the WI could be ignored.

### 3.3. Optimal Model

#### 3.3.1. Optimal Model Using Different Modeling Methods

Six models were selected from the PLS models constructed using the four aforementioned methods. Simultaneously, the optimal spectral preprocessing method and vegetation index were identified, and their cross validation and prediction accuracies were also obtained (Table 7). Among the AB-PLS, PCA-PLS, and CA-PLS models, the one established using $R^{\prime }$ had the highest accuracy. Therefore, $R^{\prime }$ was the best spectral preprocessing method. In the CA(W)-PLS models, log(1/R) was the best spectral preprocessing method. Among the vegetation index models, GNDVI was the best among the 10 vegetation indices, and WI was the best among the four water indices.

In terms of cross validation accuracy, AB-PLS, PCA-PLS, and CA(W)-PLS performed relatively better, with R^2^*_CV_* > 0.8 in all of them, whereas the remaining models had R^2^*_CV_* < 0.8. Similarly, in terms of prediction accuracy, these three models also performed relatively better. The maximum differences in cross validation accuracy indicators among them were as follows: R^2^*_CV_* = 0.0675, and RMSE*_CV_* = 0.9448. In addition, the maximum differences in prediction accuracy indicators among them were as follows: R^2^*_P_* = 0.0467, and RMSE*_P_* = 0.5221. Therefore, because the differences among the three models were not significant enough, further model selection was needed.

In the CA(W)-PLS model, the water bands contributed significantly to cross validation accuracy and should be fully considered when selecting feature bands to build the model. Compared with the CA-PLS model, the CA(W)-PLS model showed a significant improvement in cross validation accuracy, with an increase of 10.88% in R^2^*_CV_* and a decrease of 19.51% in RMSE*_CV_*. The prediction accuracy of the CA(W)-PLS model also improved to some extent, with an increase of 11.26% in R^2^*_P_* and a decrease of 20.69% in RMSE*_P_*.

In the GNDVI + WI-L model, the contribution of water factors to cross validation accuracy was negligible. Thus, the influence of the WI could be ignored when modeling using vegetation indices. Compared with the GNDVI-L model, the GNDVI + WI-L model showed a slight improvement in cross validation accuracy, with an increase of 0.0003 in R^2^*_CV_* and a decrease of 0.0030 in RMSE*_CV_*. However, the prediction accuracy of the latter declined slightly, with a decrease of 0.0006 in R^2^*_P_* and an increase of 0.0015 in RMSE*_P_*, further demonstrating that water information could be ignored.

Among the six models, the GNDVI-L model exhibited lower accuracy (R^2^ *_CV_* = 0.7624, RMSE*_CV_* = 3.3518), and its prediction accuracy was at a moderate level (R^2^*_P_* = 0.7619, RMSE*_P_* = 3.3667). Because a single-vegetation index used only two to four bands to build the model, it contained less spectral information, leading to relatively low cross validation accuracy.

#### 3.3.2. Optimal Model Based on the Leaf Position

**Figure 5:**
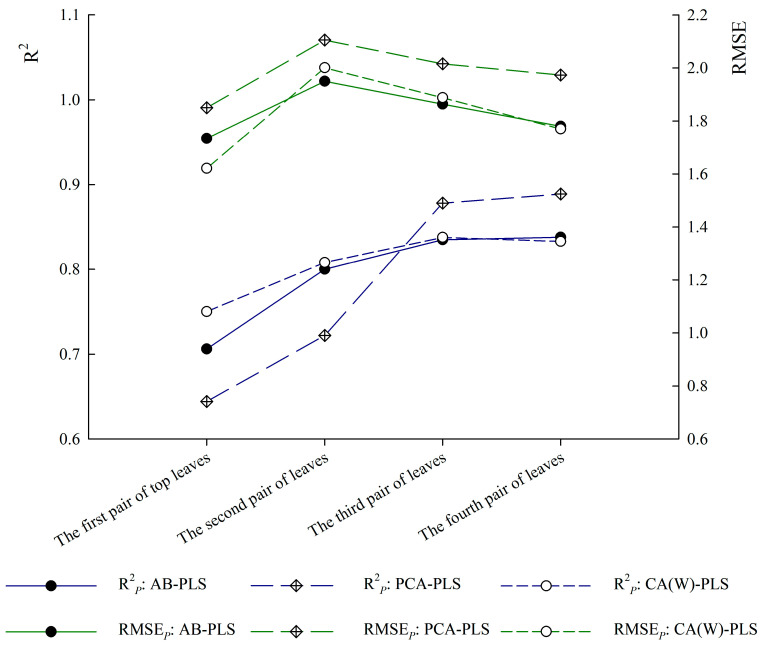
Prediction accuracy of the three optimal models at different leaf positions.

The prediction accuracy of the models varied with the leaf positions, and all three models showed the worst prediction ability for the first pair of top leaves, which was significantly lower than the other leaf positions in both cross validation accuracy and prediction accuracy (Table 7 and Figure 5). The first pair of top leaves was involved in treatments T1, T2, T3, and T4; the second pair of leaves in treatments T2, T3, and T4; the third pair of leaves in treatments T3 and T4; and the fourth pair of leaves only in treatment T4. Therefore, the first pair of top leaves was the leaf position that underwent the most complex changes. This complexity inevitably affected its prediction ability, making it perform worse in the CA(W)-PLS, AB-PLS, and PCA-PLS models. It became a bottleneck which limited prediction ability, and consequently became a prioritized criterion for selecting the optimal model over other criteria. The prediction ability of the first pair of top leaves ranked as follows: CA(W)-PLS > AB-PLS > PCA-PLS. Based on the prediction accuracy of the first pair of top leaves, CA(W)-PLS was the optimal model.

The range of prediction accuracy varied among the three models at different leaf positions, with the CA(W)-PLS model showing a relatively smaller variation (Figure 5). The range of R^2^*_P_* values varied as follows: 0.1320 for the AB-PLS model, 0.2450 for the PCA-PLS model, and 0.0877 for the CA(W)-PLS model. Similarly, the variation in RMSE*_P_* values was as follows: 0.3893 for the AB-PLS model, 0.3349 for the PCA-PLS model, and 0.3009 for the CA(W)-PLS model. Overall, the CA(W)-PLS model demonstrated consistent accuracy across the different leaf positions. Based on this consistent prediction ability across different leaf positions, the CA(W)-PLS model was the optimal model, and its modeling formula:

*C* = 2.1754 + 57.7898 × *R*_547_ + 18.9012 × *R*_548_ − 151.3200 × *R*_549_ − 103.7400 × *R*_550_ + 39.6828 × *R*_551_ + 142.0900 × *R*_552_ + 489.6450 × *R*_1464_ − 494.9750 × *R*_1465_
*−* 36.6924 × *R*_1933_ + 37.3505 × *R*_1944_ (5)

where *C* is the chlorophyll content (mg/g), *R*_547_, *R*_548_*, R*_549_, *R*_550_, *R*_551_, *R*_552_, *R*_1464_, *R*_1465_, *R*_1933_ and *R*_1944_ are the spectral reflectance at 547 nm, 548 nm, 549 nm, 550 nm, 551 nm, 552 nm, 1464 nm, 1465 nm, 1933 nm, and 1944 nm respectively.

## 4. Discussion

The choice of spectral preprocessing method directly affects the quality of the model. Using an appropriate method effectively improves cross validation accuracy, whereas an improper method can lead to a decline in model quality [10]. In this study, 23 spectral transformations were used for AB-PLS modeling. Among these, the original spectrum ranked sixth in terms of effectiveness. Seventeen methods reduced the model quality, and only five methods effectively improved the cross validation accuracy. Using six accurate spectral preprocessing methods, the AB-PLS, PCA-PLS, and CA-PLS models were established. Among these models, the one established using $R^{\prime }$ had the highest accuracy, confirming $R^{\prime }$ as the best spectral preprocessing method. Similarly, in a study modeling the chlorophyll content in peach leaves across four growth stages using five spectral transformations (R, $R^{\prime }$, $R^{\prime \prime }$, 1/R, and log(R)), R′ was identified as the optimal spectral preprocessing method [45]. In a correlation analysis, $R^{\prime }$ had the strongest correlation with the chlorophyll content in the six spectral preprocessing methods [46]. The strongest correlation itself can also explain to some extent why $R^{\prime }$ was identified as the best spectral preprocessing method. In the CA(W)-PLS models, the cross validation accuracies of log R, log (1/R), and log R were nearly consistent, with log (1/R) narrowly ranking as the best spectral preprocessing method. In predicting the chlorophyll content in eucalyptus leaves using models built using four spectral transformations (R, $R^{\prime }$, log(1/R), and $R^{\prime \prime }$), and feature bands selected through correlation analysis, log(1/R) was identified as the best spectral preprocessing method [15]. Among the CA-PLS models, $R^{\prime }$ was the best spectral preprocessing method, whereas among the CA(W)-PLS models, log(1/R) was optimal. These findings indicate that changing the feature band can affect the selection of the best spectral preprocessing method. When predicting the chlorophyll content in *maple* trees based on a stepwise regression using four spectral transformations (R, $R^{\prime }$, log(1/R), and ${\left(1/logR\right)}^{\prime }$), the best spectral preprocessing method identified was ${\left(1/logR\right)}^{\prime }$ [4]. In this study, six spectral transformations combined with the PLS models were screened, and log(1/R) was proven to be the best preprocessing method. These results indicate that different prediction models could lead to different optimal preprocessing methods. When predicting the chlorophyll content in *wasabi* leaves using five preprocessing methods and five machine learning algorithms, gaps were observed in the prediction accuracy when different preprocessing methods were combined with different prediction models to estimate the chlorophyll content [11]. This also illustrated that the model construction method affected the selection of the best spectral preprocessing method.

The reflectivity of *Paulownia* seedling leaves was affected by the chlorophyll and water content, and drought stress caused significant changes. The curve of the average spectral reflectance showed that, under drought stress, the sensitive band of chlorophyll content was located at the reflection peak of green light near 550 nm, and the sensitive band of water content was near 1440 and 1920 nm. In this study, the influence of water factors on the model was considered when establishing the prediction model. AB-PLS used all-band direct modeling, and PCA-PLS used all-band dimensionality reduction modeling, with both models incorporating water bands. The CA-PLS model became the CA(W)-PLS model after the water feature band was added, resulting in a significant improvement in the cross validation accuracy and prediction accuracy. This indicates that considering the water-sensitive band can improve the prediction accuracy of the chlorophyll content [13]. Traditional studies of plant physiology and biochemistry have established a correlation between the chlorophyll content and water content in plant leaves under drought stress [47]. This indicated that it was appropriate to take water bands into consideration in predicting the chlorophyll content in *Paulownia* seedling leaves. After adding WIs, the model quality of both multiple-index and single-index models changed slightly. On the one hand, the cross validation accuracy of all the models slightly increased; on the other hand, the prediction accuracy of GNDVI-L was slightly higher than that of GNDVI + WI-L, which implied that water factors slightly reduced the prediction ability. In conclusion, the influence of WI was very small, and could be either negative or positive. Therefore, there is no need to consider the influence of water bands when modeling with vegetation indices.

Vegetation index modeling showed that, as the number of indicators increased, the cross validation accuracy increased. When using different numbers of vegetation indices to build a model for chlorophyll content in rapeseed leaves, the cross validation accuracy of the multi-vegetation index models was found to be significantly higher than that of the models using fewer vegetation indices and was much higher than that of a single-vegetation index model [48]. Similarly, when building a model to estimate the chlorophyll content in cotton leaves, the accuracy of the multi-vegetation index models was higher than that of the models built using fewer vegetation indices [49]. Single-vegetation indices are usually constructed using two to four selected bands, which contain limited spectral information, thereby resulting in relatively low cross validation accuracy. However, because of their simplicity, the predicted and measured values match well. Therefore, vegetation index modeling is one of the most commonly used methods. Vegetation indices vary in accuracy for estimating chlorophyll content; although they are all sensitive to chlorophyll, they focus on different aspects and contain different spectral information. Multi-vegetation index models incorporate multiple vegetation indices, which increases the amount of chlorophyll-sensitive spectral information available, thereby improving cross validation accuracy. As the number of indicators increases, the accuracy of the cross validation also increases. However, the accuracy of the vegetation index model is generally lower than that of the all-band direct and the all-band dimensionality reduction models. The accuracies of the 10VI-PLS and 10VI + 4WI-PLS models were lower than those of the AB-PLS and PCA-PLS models, consistent with the findings from a study on *Pterocarya stenoptera* seedlings [8].

Chlorophyll content in plant leaves exhibits a vertical distribution [7,50], and hence leaf positions are bound to become influencing factors in prediction model research. Moreover, whether this factor is considered can significantly affect the selection of the optimal model. In a study on hyperspectral prediction models for chlorophyll content in healthy winter wheat leaves, the cross validation accuracy of PLS at different leaf positions was as follows: second leaf > flag leaf > third leaf [7]. In this study, the hyperspectral prediction of chlorophyll content in the leaves of *Paulownia* seedlings under drought stress was investigated. The results revealed that the prediction ability of the first pair of top leaves was the lowest. The difference in the results may be attributed to the vertical distribution of the chlorophyll content in the leaves of *Paulownia* seedlings under the same drought treatment, which was further complicated by different drought treatments. In addition, the first pair of top leaves was involved in four different drought treatments, and therefore underwent the most complex changes. In the T1 treatment, the first pair of top leaves were almost falling off due to drought, whereas in the T4 treatment, the first pair of top leaves were healthy young leaves. In this study, without considering the prediction accuracy of leaf positions, AB-PLS was identified as the optimal modeling method. In terms of cross validation accuracy, the ranking was PCA-PLS > AB-PLS > CA(W)-PLS. In terms of prediction accuracy, the ranking was AB-PLS > CA(W)-PLS > PCA-PLS (Table 7). Regarding the prediction accuracy across leaf positions, the first pair of top leaves exhibited the poorest performance and were significantly lower than that of other leaf positions in terms of both cross validation accuracy and prediction accuracy. Given this prediction bottleneck, CA(W)-PLS was the optimal model.

## 5. Conclusions

Based on 23 spectral preprocessing methods and four PLS model construction methods, considering the influence of water bands on prediction accuracy and their uniformity across different leaf positions, the optimal model for predicting chlorophyll content was systematically explored. This study ensured the systematicity, applicability, and stability of the model and provided a practical and feasible approach for the quantitative inversion of chlorophyll content in *Paulownia* leaves under drought stress.

(1) A specific approach to constructing PLS models corresponds to a certain optimal spectral preprocessing method. Among the AB-PLS, PCA-PLS, and CA-PLS models, $R^{\prime }$ was identified as the optimal spectral preprocessing method. For the CA(W)-PLS model, log(1/R) was identified as the optimal spectral preprocessing method. Among the 14 selected vegetation indices, GNDVI was preferred, and WI was the preferred water index.

(2) Water bands affected cross validation accuracy. When selecting feature bands for modeling, considering the influence of water feature bands is crucial, as they significantly increased cross validation accuracy. When modeling with vegetation indices, water indices slightly increased the cross validation accuracy but reduced predictive ability to a negligible extent. 

(3) CA(W)-PLS emerged as the optimal model. The first pair of top leaves, which exhibited the most complex changes, was a bottleneck that limited the predictive power of the model because the prediction accuracy was significantly lower than that of the other leaf positions in terms of both cross validation accuracy and predicting accuracy. Based on the prediction accuracy of the first pair of top leaves and the uniformity of prediction accuracy across the different leaf positions, the CA(W)-PLS model was identified as the optimal model.

## Figures and Tables

**Table 1:** AB-PLS cross validation accuracy using the six spectral preprocessing methods.

Spectral Transformation	R^2^*_CV_*	RMSE*_CV_*
$R^{\prime }$	0.8329	2.6422
$\sqrt{R}$	0.8317	2.6566
log⁡R	0.8295	2.6829
log(1/R)	0.8295	2.6829
$log\sqrt{R}$	0.8295	2.6829
R	0.8268	2.7154

**Table 2:** PCA-PLS cross validation accuracy using the six spectral preprocessing methods.

Spectral Transformation	Cumulative Variance Contribution Rate/(%)	Number of Principal Components	R^2^*_CV_*	RMSE*_CV_*
$R^{\prime }$	96.08%	170	0.8769	1.9637
$\sqrt{R}$	99.83%	9	0.8338	2.6310
log⁡R	99.82%	9	0.8354	2.6109
log(1/R)	99.82%	9	0.8354	2.6109
$log\sqrt{R}$	99.82%	9	0.8354	2.6109
R	99.87%	10	0.8293	2.6857

**Table 3:** Correlation analysis of spectral transformation and chlorophyll content.

Spectral Transformation	Correlation Analysis		Correlation Analysis (Water Band)	
	Feature Band Selection/nm	Correlation Coefficient	Feature Band Selection/nm	Correlation Coefficient
$R^{\prime }$	744, 742, 743, 741, 745, 740, 739, 747, 746, 738	0.8124, 0.8120, 0.8118, 0.8088, 0.8067, 0.8045, 0.8041, 0.8026, 0.8001, 0.7981	744, 742, 743, 741, 745, 740, 1464, 1465, 1933, 1944	0.8124, 0.8120, 0.8118, 0.8088, 0.8067, 0.8045, 0.0167, −0.0166, 0.0756, 0.0800
$\sqrt{R}$	550, 551, 549, 548, 552, 547, 546, 553, 545, 544	−0.7925, −0.7925, −0.7925, −0.7924, −0.7924, −0.7924, −0.7923, −0.7923, −0.7922, −0.7921	550, 551, 549, 548, 552, 547, 1464, 1465, 1933, 1944	−0.7925, −0.7925, −0.7925, −0.7924, −0.7924, −0.7924, 0.0767, 0.0768, 0.0832, 0.0847
log⁡R	550, 551, 549, 548, 547, 552, 546, 545, 553, 544	−0.7908, −0.7908, −0.7908, −0.7908, −0.7907, −0.7907, −0.7907, −0.7906, −0.7905, −0.7905	550, 551, 549, 548, 547, 552, 1464, 1465, 1933, 1944	−0.7908, −0.7908, −0.7908, −0.7908, −0.7907, −0.7907, 0.0743, 0.0744, 0.0632, 0.0669
log⁡(1/R)	550, 551, 549, 548, 547, 552, 546, 545, 553, 544	0.7908, 0.7908, 0.7908, 0.7908, 0.7907, 0.7907, 0.7907, 0.7906, 0.7905, 0.7905	550, 551, 549, 548, 547, 552, 1464, 1465, 1933, 1944	0.7908, 0.7908, 0.7908, 0.7908, 0.7907, 0.7907, −0.0743, −0.0744, −0.0632, −0.0669
$log\sqrt{R}$	550, 551, 549, 548, 547, 552, 546, 545, 553, 544	−0.7908, −0.7908, −0.7908, −0.7908, −0.7907, −0.7907, −0.7907, −0.7906, −0.7905, −0.7905	550, 551, 549, 548, 547, 552, 1464, 1465, 1933, 1944	−0.7908, −0.7908, −0.7908, −0.7908, −0.7907, −0.7907, 0.0743, 0.0744, 0.0632, 0.0669
R	550, 551, 549, 552, 548, 547, 553, 546, 545, 554	−0.7890, −0.7890, −0.7890, −0.7890, −0.7889, −0.7889, −0.7888, −0.7888, −0.7886, −0.7886	550, 551, 549, 552, 548, 547, 1464, 1465, 1933, 1944	−0.7890, −0.7890, −0.7890, −0.7890, −0.7889, −0.7889, 0.0819, 0.0819, 0.1052, 0.1052

**Table 4:** CA-PLS and CA(W)-PLS cross validation accuracy using the six spectral preprocessing methods.

Spectral Transformation	CA-PLS		CA(W)-PLS	
	R^2^*_CV_*	RMSE*_CV_*	R^2^*_CV_*	RMSE*_CV_*
$R^{\prime }$	0.7300	3.6133	0.7566	3.4001
$\sqrt{R}$	0.6963	3.8607	0.7967	3.0373
log⁡R	0.6933	3.8817	0.8093	2.9086
log⁡(1/R)	0.6933	3.8817	0.8094	2.9085
$log\sqrt{R}$	0.6933	3.8817	0.7862	3.1384
R	0.6901	3.9042	0.7937	3.0666

**Table 5:** Correlation analysis of spectral index and chlorophyll content.

Spectral Index	Algorithm Formula	Reference	Correlation Coefficient
Green normalized difference vegetation index (GNDVI)	$GNDVI=\left(R_{750}-R_{550}\right)/\left(R_{750}+R_{550}\right)$	[32]	0.8286
Chlorophyll index at green band (CI_green_)	${CI}_{green}=\left(R_{800}/R_{550}\right)-1$	[33]	0.8186
Ratio vegetationindex (RVI)	$RVI=R_{810}/R_{570}$	[34]	0.8012
Chlorophyll index at red edge band (CI_red edge_)	${CI}_{red\;edge}=\left(R_{800}/R_{720}\right)-1$	[33]	0.7920
Vogelmann red edge index 3 (VOG3)	$VOG3=(R_{734}-R_{747})/({R_{715}+R}_{720})$	[35]	−0.7775
Red-edge normalized difference vegetation index (RNDVI)	$RNDVI=(R_{750}-R_{705})/({R_{750}+R}_{705})$	[36]	0.7504
Normalized difference vegetation index (NDVI)	$NDVI=(R_{800}-R_{670})/({R_{800}+R}_{670})$	[37]	0.5253
photochemical reflectance index (PRI)	$PRI=(R_{531}-R_{570})/({R_{531}+R}_{570})$	[38]	0.4358
Normalized pigment chlorophyll index (NPCI)	$NPCI=(R_{680}-R_{430})/({R_{680}+R}_{430})$	[39]	−0.3153
Triangular vegetation index (TVI)	$TVI=60\left(R_{750}-R_{500}\right)-100\left(R_{670}-R_{500}\right)$	[40]	0.2424
Water index (WI)	$WI=R_{900}/R_{970}$	[41]	−0.2151
Water band index (WBI)	$WBI=R_{950}/R_{900}$	[42]	0.2116
Normalized difference water index (NDWI)	$NDWI=(R_{860}-R_{1240})/({R_{860}+R}_{1240})$	[43]	−0.1613
Moisture stress index (MSI)	$MSI=R_{1600}/R_{820}$	[44]	0.1417

**Table 6:** Cross validation accuracy using the spectral indices.

Model	R^2^*_CV_*	RMSE*_CV_*	Model	R^2^*_CV_*	RMSE*_CV_*
10VI-PLS	0.8194	2.8000	10VI + 4WI-PLS	0.8246	2.7410
6VI-PLS	0.7984	3.1352	6VI + 4WI-PLS	0.7989	3.0977
GNDVI-L	0.7624	3.3518	GNDVI + WI-L	0.7627	3.3488
GI_green_-L	0.7438	3.5056	GI_green_ + WI-L	0.7438	3.5055
RVI-L	0.7121	3.7476	RVI + WI-L	0.7121	3.7472

**Table 7:** Optimal model using different modeling methods.

Model	Model Index	Cross Validation Accuracy		Predicted Accuracy	
		R^2^*_CV_*	RMSE*_CV_*	R^2^*_P_*	RMSE*_P_*
AB-PLS	$R^{\prime }$	0.8329	2.6422	0.8503	2.4567
PCA-PLS	$R^{\prime }$	0.8769	1.9637	0.8036	2.9788
CA-PLS	$R^{\prime }$	0.7300	3.6133	0.7272	3.6519
CA(W)-PLS	log⁡(1/R)	0.8094	2.9085	0.8091	2.8963
GNDVI-L	GNDVI	0.7624	3.3518	0.7619	3.3667
GNDVI + WI-L	GNDVI, WI	0.7627	3.3488	0.7613	3.3682

## Data Availability

Please contact the corresponding authors.
